# DNA Synthesis Is Activated in Mosquitoes and Human Monocytes During the Induction of Innate Immune Memory

**DOI:** 10.3389/fimmu.2018.02834

**Published:** 2018-11-30

**Authors:** Jorge Cime-Castillo, Rob J. W. Arts, Valeria Vargas-Ponce de León, Ramon Moreno-Torres, Salvador Hernández-Martínez, Benito Recio-Totoro, Fabiola Claudio-Piedras, Mihai G. Netea, Humberto Lanz-Mendoza

**Affiliations:** ^1^Centro de Investigaciones Sobre Enfermedades Infecciosas, Instituto Nacional de Salud Pública, Cuernavaca, Mexico; ^2^Department of Internal Medicine and Radboud Center for Infectious Diseases, Radboud University Medical Center, Nijmegen, Netherlands; ^3^Facultad de Estudios Superiores Iztacala, Universidad Nacional Autonoma de México, Mexico City, Mexico; ^4^Department for Genomics and Immunoregulation, Life and Medical Sciences Institute, University of Bonn, Bonn, Germany

**Keywords:** endoreplication, DNA synthesis, trained immunity, monocytes, mosquitoes

## Abstract

Endoreplication is a cell cycle program in which cells replicate their genomes without undergoing mitosis and cytokinesis. For the normal development of many organisms (from fungi to humans) and the formation of their organs, endoreplication is indispensable. The aim of the present study was to explore whether endoreplication and DNA synthesis are relevant processes during the induction of trained innate immunity in human monocytes and in the *Anopheles albimanus* mosquito cell line. During the induction of trained immunity in both models, endoreplication markers were overexpressed and we observed an increase in DNA synthesis with an augmented copy number of genes essential for trained immunity. Blocking DNA synthesis prevented trained immunity from being established. Overall, these findings suggest that DNA synthesis and endoreplication are important mechanisms involved in inducing innate immune memory. They have probably been conserved throughout evolution from invertebrates to humans.

## Introduction

Both old and recent studies have demonstrated that innate immunity is able to adapt in vertebrates, invertebrates, and plants upon encountering an infection, representing a *de facto* innate immune memory [see review for invertebrates (1), plants (2), and vertebrates (3, 4)]. Such an adaptation is denominated trained immunity (TI) or innate immune priming (IP). Although the molecular mechanisms underlying TI are not completely understood, epigenetic reprogramming reportedly plays a relevant role in human monocytes (5). Monocytes can be trained by pre-exposure to β-glucan of *Candida albicans* or the BCG vaccine, leading in both cases to an enhanced and long-lasting response to microbial components at a later time (6–8). This is the result of epigenetic histone changes at the level of H3K27ac and H3K4me3 (5).

Epigenetic reprogramming is also known to participate in systemic acquired resistance, a process of epigenetic-induced immune memory in plants (2). These epigenetic changes can be transferred to the progeny of plants through their seeds. For plant immune memory, the acetylation of H3K9 is key.

Interesting evidence was recently presented on the molecular mechanisms of immune priming or innate immune memory in invertebrates. For the insect *Tenebrio molitor*, adults were primed against the bacteria *Micrococcus lysodeikticus* and larvae against the fungus *Metarhizium anisopliae*. Afterwards, a reduction in methylated cytosine entities was found in RNA (5 mC) within several generations (9). In the planaria *Schmidtea mediterranea*, on the other hand, primo-infection gives rise to the expression of the peptidoglycan receptor Smed-PGRP-2, which promotes Smed-setd8-1 histone methyltransferase expression and thus increases the level of lysine methylation in histones (10).

Now known as a critical mechanism of TI, epigenetic reprogramming allows cells to rapidly produce sufficient proteins to adequately respond to a second challenge. Other likely mechanisms that could account for this augmented immune response are DNA synthesis and endoreplication. Endoreplication refers to multiple rounds of nuclear genome duplications that do not result in nuclear division and cytokinesis (11). Endoreplication is thought to occur in response to cellular stress (12), particularly in highly specialized, differentiated cells that generate large amounts of RNA and proteins (13). Endoreplication has been observed in plant development, flowering plants, mollusks, arthropods, amphibians, fish, and mammals (14). Cardiac myoblast, basal epithelial cells, and primitive podocytes endoreplicate in mice when they are under stress, as in injury or infection (15). In humans, endoreplication has been identified in megakaryocytes, hepatocytes, cardiomyocytes, the endometrium, and the epidermis (16–20).

We previously reported that *de novo* DNA synthesis occurs in *Anopheles albimanus* mosquitoes following an immune challenge (21, 22), as shown by the incorporation of bromodeoxyuridine (BrdU), the formation of polytene chromosomes, and the activation of proliferating cell nuclear antigen (PCNA) in the tissues of these insects. Such synthesis is apparently by endoreplication, as no mitotic cells have been detected. In another study by our group, enhanced DNA synthesis was observed in the midgut of *An. albimanus* mosquitoes after the second exposure to the same pathogen (23), and more recently in *Aedes aegypti* mosquitoes treated with the dengue virus (24, 25). In the latter effort, we also explored the role played by one of the key elements, hindsight (*HNT*, a zinc-finger transcription factor), in cell cycle switching toward endoreplication. In all eukaryotic cells and in *Drosophila, HNT* is involved in mediating the participation of the Notch pathway in the switching of the cell cycle from mitosis to the endocycle (26, 27).

The aim of the present study was to analyze the role of DNA synthesis (endoreplication) during the establishment of TI in human monocytes and an *Anopheles albimanus* mosquito cell line. In both these models, a first challenge induced DNA synthesis (evidenced by the incorporation of BrdU), an increased concentration of DNA and the overexpression of *HNT*. With the blocking of DNA synthesis, the expression of immune memory markers was found to be at the level of the control cells, indicating that TI was inhibited.

## Materials and methods

### Cell culture

LSB-AA695BB is a cell line obtained from embryos of *Anopheles albimanus* mosquitoes (28). Cells were propagated in 24-well plates (Corning) in supplemented Schneider medium (10% fetal bovine serum; FBS Byproducts). When cells reached 80% confluence, they were depleted of FBS and challenged with *Plasmodium berghei* ookinetes (98% purity), as described by Recio-Totoro et al. (in preparation). Following purification, these parasites were killed through three freeze-thaw cycles, centrifuged at 5,000 rpm for 10 min and resuspended in Schneider medium. Parasite concentration per mL was calculated in a Neubauer chamber. The challenge to an average of 5 × 10^4^ cells per well were carried out with 96,000 ookinetes. At 1, 3, or 6 h of the challenge, parasites were removed from the cells. To induce TI, a well was challenged as aforementioned and the inoculum was withdrawn after 6 h of infection. Seven days later, the cells were again exposed, but to only half (48,000) the quantity of the parasites employed in the prior challenge. They were left for 6 h before removing the inoculum.

Cell lines were incubated with Zymosan (β-1,3-glucan, Sigma-Aldrich) at a concentration of 1 μg/μL for 1, 3, and 6 h. In additional experiments, cells were challenged with Zymosan or *Plasmodium berghei* ookinetes in the presence of cisplatin (Sigma-Aldrich) at a concentration of 1 μM at the designated times. Upon completion of the corresponding time, cells were washed three times with PBS to remove the stimulus and subsequently harvested for the extraction of genetic material. As the control, unchallenged cells were included and taken at each of the harvest time points.

### Obtaining genetic material and synthesis of cDNA

Challenged cells and control cells were lysed with RIPA buffer and suspended in 2 mL of Schneider medium. A cell counter was used to verify the number and viability of cells (Countess, Invitrogen), finding an average of 95% cell viability. The quantity of cells was adjusted to 1 × 10^4^ before beginning RNA extraction, which was carried out with TRIzol (Invitrogen). Following the recommendations of the supplier, the retrieved RNA was solubilized in 30 μL of water free of nucleases (H_2_O_Dpec_) and stored at −70°C.

The cDNA was synthesized for each of the samples, normalizing it to 1 μg of RNA_total_. Briefly, in a 200 μl-microtube a mix was prepared of 500 ng/μg RNA_total_, 1 μl of random hexamers at 50 μg/ml (Thermo Scientific), and 1 μl of 10 mM dNTPs. After the mixture was adjusted to a volume of 10 μl with H_2_O_Depc_, the samples were incubated at 65°C for 5 min. Then 2 μl of Buffer 5 X (Thermo Scientific), 0.5 μl of RNase inhibitor at 40 U/μl (RiboLock RNase Inhibitor; Thermo Scientific), and 0.5 μl reverse transcriptase at 200 U/μl (RevertAid reverse; Thermo Scientific) were added. The reaction mixture was adjusted to a final volume of 10 μl with H_2_O_Dpec_. Samples were incubated at 25°C for 10 min, 37°C for 60 min and 70°C for 10 min in a thermocycler (T100 Thermal cycle; Bio-Rad).

For the quantification of DNA by cells, genetic material was obtained from a cell culture prepared in a 24-well plate at 3 and 6 h of the challenge with *Plasmodium berghei* (or unchallenged control cells). Prior to the extraction of the DNA, cell viability of 62,500, 125,000, 250,000, and 500,000 cells in control and experimental conditions was verified by a cell counter (Invitrogen) and in trypan blue dye exclusion. Genetic material extraction was performed with DNazol reagent (Thermo Scientific), following the indications of the suppliers. To avoid the presence of RNA in the samples, the homogenate was centrifuged for 10 min at 10,000 g and at 4°C, and then the supernatant was transferred to a fresh tube. This step removes insoluble tissue fragments, RNA and excess of polysaccharides from the lysate/homogenate. Additionally, RNase A [1 ug/ml] was added to the mixture before the PCR assay. Subsequently, the amount of DNA was measured with NanoDrop (Thermo Scientific).

### Quantification by real-time PCR

The amplification of genes of the *An. albimanus* immune response was carried out with previously recovered genetic material. Specific primers were used for each gene (Table 1). The genes were chosen because they are involved in the mosquito immune response against *Plasmodium*.

**Table 1:** Primer sequence and length of the amplified product.

**Gene**	**Forward primer**	**Reverse primer**	**Cells**	**Product size**
*CTL4*	CAATCGCAAAATACAGCTCGTG	CCAGTAGGACGAGGAACGAAG	Mosquito	231 Bp
*CTL6*	CTGGATGCGTACTTTGAATGC	CAAAGGTCCTCTTTGCGATCA	Mosquito	116 Bp
*HNT*	CGTAGTGCCTGTCCCAAACT	ATTGTTGCCGCTGCTCT	Mosquito	125 Bp
*TEP1*	GTGAACTTGCCGAGTGGCTA	CGACAGTAGTACCACCGTAGAGG	Mosquito	106 Bp; 185 Bp^*^
*PPO1*	GGCGGACCAAATCAAGCAG	CGATTGCCCGATTCGTCAAC	Mosquito	102 Bp; 185 Bp^*^
*LRIM1*	CGTGCTCGCTAGCTACGTT	CGTAGTGCCTGT CCCAAACT	Mosquito	117 Bp
*HK2*	GAGCTCAATTCTGTGTGGAGT	ACTTCTTGAGAACTATGTACCCTT	Monocytes	77 Bp
*PFKP*	CGAAGGCGATGGGGTGAC	CATCGCTTCGCCACCTTTC	Monocytes	75 Bp
*TNFA*	GTGCTTGTTCCTCAGCCTCT	ATCACTCCAAAGTGCAGCAG	Monocytes	81 Bp
*IL6*	AGGGAGAGCCAGAACACAGA	GAGTTTCCTCTGACTCCATCG	Monocytes	97 Bp

CTL4, C-type lectin 4; CTL6, C-type lectin 6; HNT, Hindsight, TEP-1, thioester-containing protein 1; PPO1, prophenoloxidase 1; LRIM1, leucine-rich immune protein; HK2, hexokinase 2; PFKP, phosphofructokinase; TNFA, tumor necrosis factor; IL6, interleukin 6.

* *Amplified product length in the genomic sequence. The primers were designed between two exons to reveal the genomic product and the product of the transcript in DNA synthesis as well as the gene expression level*.

The samples were run in a real-time thermal cycler (viiA7; Applied Biosystems) under optimal running conditions, according to the manufacturer's recommendations. Samples were incubated at 60°C in a master mix containing Sybr Green (Maximum SYBR Green/Rox qPCR Master Mix; Thermo Scientific), primers, and cDNA of each of the samples, set to a volume of 20 μl with water free of nucleases (Thermo Scientific). The relative expression was quantified by employing the ΔΔCt method, normalizing expression of immune response genes with the S7 ribosomal gene.

Assays were performed three times in different batches of cell culture. The control and experimental tests were made at the same time. Data from all quantitative assays were subjected to the Shapiro-Wilk normality test and then analyzed with a non-parametric Wilcoxon rank-sum test, considering significant difference at *p* < 0.05. Analyses were carried out and graphs made on GraphPad Prism v6.01.

### Trained immunity in human monocytes

Buffy coats from healthy donors were obtained after written informed consent (Sanquin blood bank, Nijmegen, The Netherlands). Isolation and stimulation was carried out as previously described (7, 29). Briefly, peripheral blood mononuclear cells (PBMCs) were isolated by density centrifugation of Ficoll-Paque (GE healthcare, UK). Cells were washed twice in PBS and resuspended in RPMI culture medium (Roswell Park Memorial Institute medium; Invitrogen, CA, USA) supplemented with 50 μg/mL gentamicin, 2 mM Glutamax (Gibco), and 1 mM pyruvate (Gibco). Percoll isolation of monocytes was performed as reported (30). Briefly, 150–200·10^6^ PBMCs were layered on top of a hyper-osmotic Percoll solution (48.5% Percoll, 41.5% sterile H_2_O, and 0.16 M filter-sterilized NaCl) and centrifuged for 15 min at 580 g. The interphase layer was isolated and cells were washed with cold PBS. For counting, cells were resuspended in RPMI culture medium supplemented with 50 μg/ml gentamicin, 2 mM glutaMAX, and 1 mM pyruvate.

Monocytes were adjusted to 1 × 10^6^ cells/mL. A 100 μL volume was added to flat-bottom 96-well plates (Corning) and cells were incubated at 37°C for 1 h. Then wells were washed once with 200 μL warm PBS to remove non-adherent cells. Subsequently, monocytes were incubated in culture medium only (negative control) or 1 μg/mL β-glucan [β-1,3-(D)-glucan, kindly provided by Professor David Williams, College of Medicine, Johnson City, USA] for 24 h. Cells were washed once with 200 μL warm PBS and incubated for 5 days in culture medium supplemented with 10% human pooled serum. The medium was changed once on day 3 of incubation. On day 6, cells were re-stimulated for 24 h in culture medium or 10 ng/mL *Escherichia coli* LPS (serotype 055:B5, Sigma-Aldrich). Afterwards, supernatants were collected and stored at −20°C until cytokine concentrations were quantified. For the inhibition experiments, 2.5 μM of cisplatin was added during the first 24 h of incubation. Cytokine production was determined in supernatants by utilizing the commercial ELISA kits for human TNFα and IL-6, according to the manufacturer's instructions (R&D systems).

For experiments with BrdU incorporation, 6 million monocytes were seeded in 10-cm Petri dishes (Corning) and treated as with β-glucan, but in the presence or absence of BrdU (Sigma-Aldrich). On day 6, cells were isolated and fixed with 1% formaldehyde. Preparations of 1 million fixed cells were processed on a Diagenode Bioruptor Pico sonicator by using five cycles of 30 s on and 30 s off. Then 12 μl of protease inhibitor cocktail, 1 μg of BrdU antibody (Sigma-Aldrich) and Protein A/G magnetic beads were added to reach a final volume of 300 μl and incubated overnight at 4°C with rotation. The next day the beads were washed with 400 μl PBS for 5 min at 4°C, subject to five rounds of washes and centrifugation. Subsequently, the pulled down beads containing BrdU-incorporated DNA were processed with 200 μl elution buffer for 20 min. Supernatant containing DNA with BrdU was collected. qPCR analysis was carried out with the corresponding primers (see Table 1). Relative expression was calculated on the RPMI/BrdU sample set, using 1 as the reference.

## Results

### DNA synthesis in the LSB-AA695BB cell line and human monocytes

**Figure 1:**
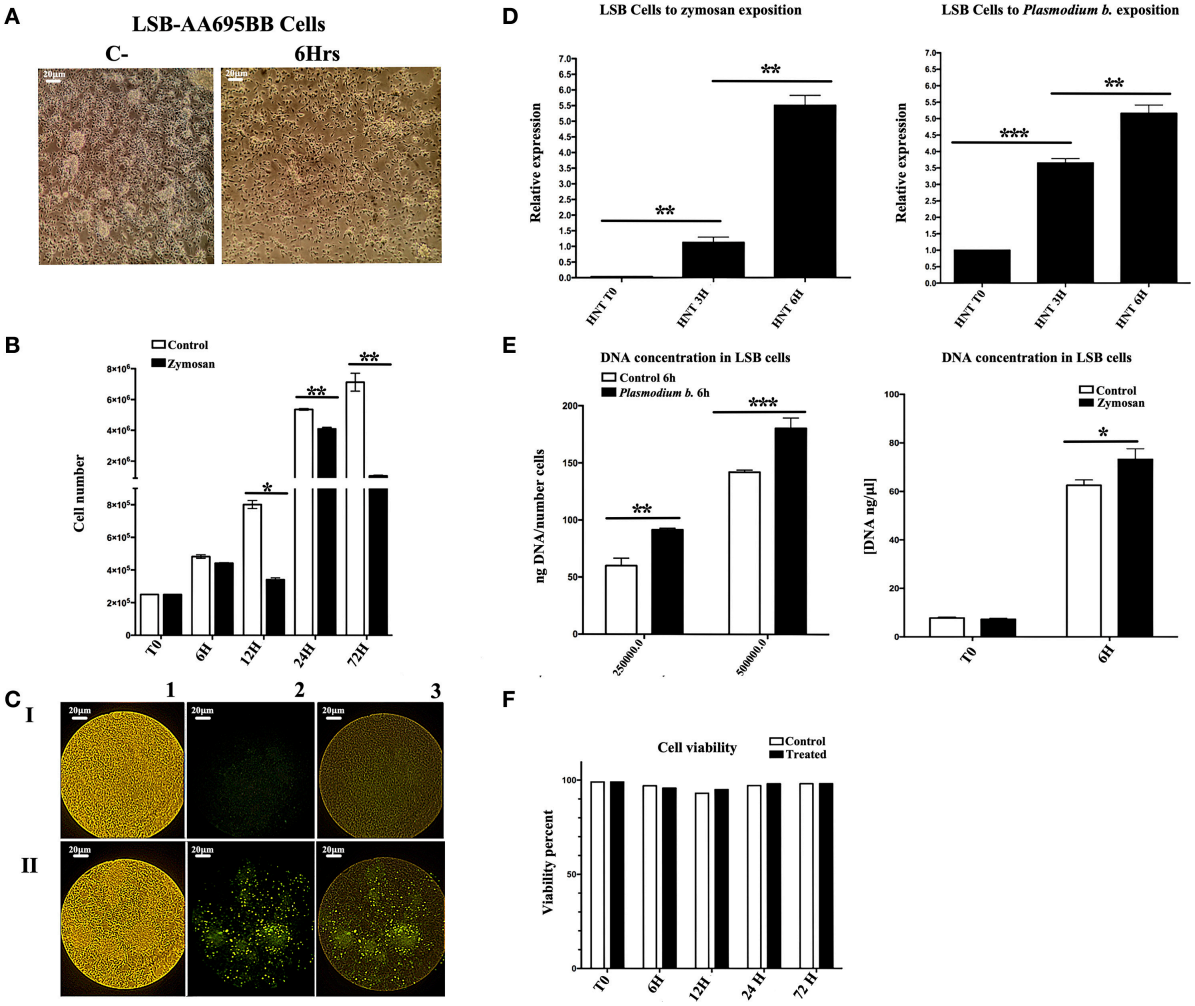
**(A)** The *Anopheles albimanus* cell line following 3 h exposure to *Plasmodium berghei* (cells without treatment to the left), 20x magnification, scale bar = 20 μm. **(B)** The number of cells in the experimental and control groups (with and without exposure to Zymosan, respectively) were determined after 6, 12, 24, and 72 h. *N* = 3, Wilcoxon; ^*^p < 0.05; ^**^p < 0.01. Values are the mean of three experiments. **(C)** The incorporation of BrdU was notable for LSB-AA695BB cells treated with Zymosan for 6 h (II), but not for untreated LSB control cells (I). The fluorescence intensity of BrdU incorporation is shown (2), as well as the merge image of BrdU and LSB cells (3). 20x magnification, scale bar = 20 μm. **(D)** Relative expression levels (ΔΔCt) of *HNT* in LSB-AA695BB cells at 0, 3, and 6 h of contact with *Plasmodium berghei or* Zymosan. *N* = 3, Wilcoxon; ^*^p < 0.05; ^**^p < 0.01. The data represent three experiments. **(E)** DNA concentration (ng/μl; ng DNA/fixed number of cells) in LSB cells following 6 h exposure to Zymosan or *Plasmodium berghei*. *N* = 3, Wilcoxon; ^*^p < 0.05; ^**^p < 0.01; ^***^p < 0.001. Values are the mean of three experiments. **(F)** Cell viability, determined by trypan blue staining in cells after 6, 12, 24, and 72 h of contact with Zymosan or *Plasmodium berghei*. The data are based on three experiments.

Endoreplication is characterized by genome duplication without cell division or cytokinesis. In order to test for a possible role of endoreplication in the mosquito cell line during the establishment of TI, cells were cultivated in the presence of Zymosan or the *P. berghei* extract. The mosquito cell line showed cellular arrest after 6 h of either of the two treatments (Figure 1A). Treated and control groups were both set at 2 × 10^5^ cells/well. Control cells, cultured without Zymosan, underwent faster duplication than Zymosan-treated cells (Figure 1B). Interestingly, BrdU incorporation (which only occurs during DNA synthesis) was elevated in cells exposed to Zymosan or the parasite extract, suggesting that endoreplication had been stimulated (Figure 1C). *HNT* gene expression, essential for the switch to endoreplication (26), and the concentration of DNA were both upregulated (vs. the control) in cells treated with Zymosan or the *Plasmodium* parasite extract (Figures 1D,E). Cell viability was maintained at around 98% in all experiments (Figure 1F).

**Figure 2:**
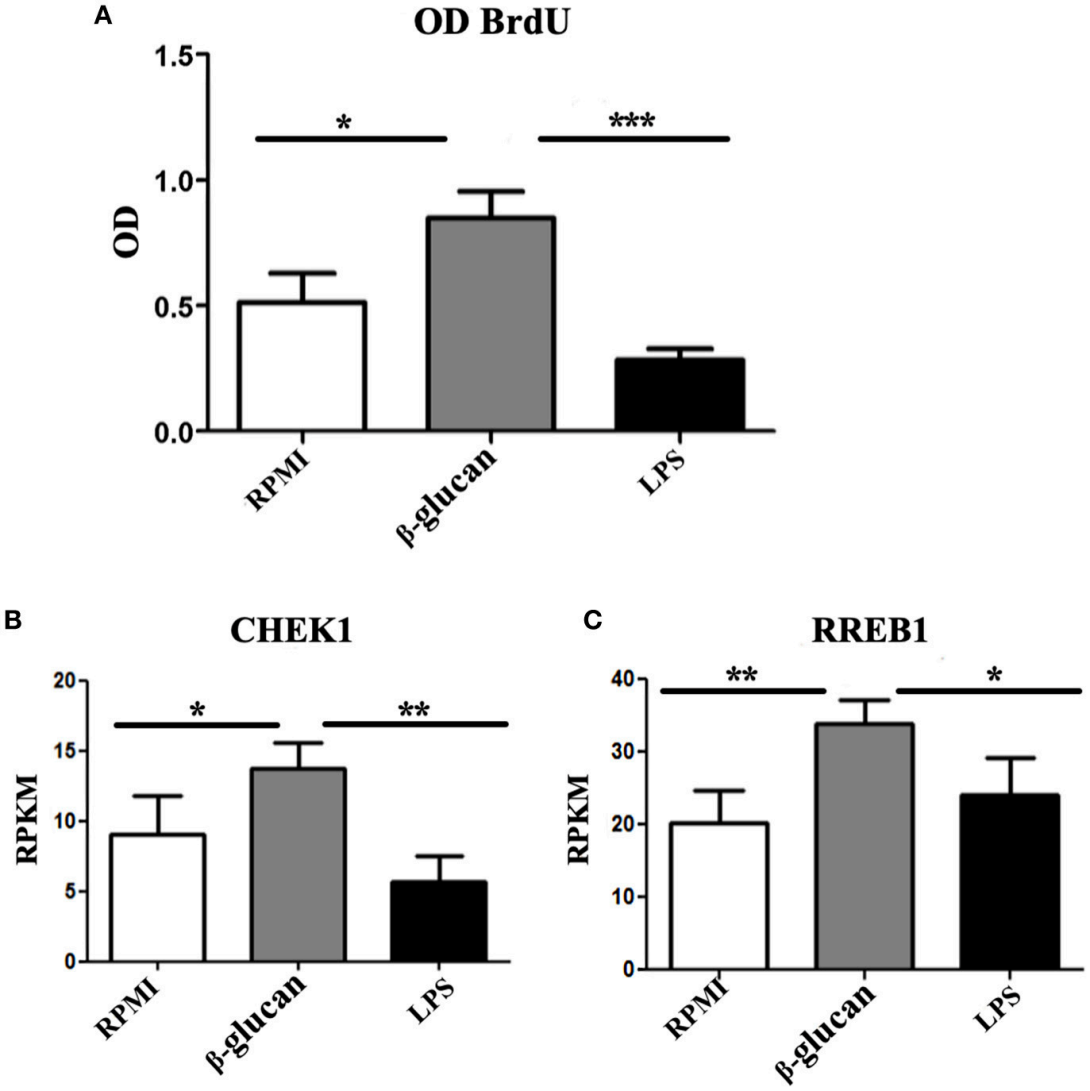
**(A)** Human monocytes were incubated for 24 h in RPMI, β-glucan or LPS. Whereas, the former was the control, the latter two treatments represent the trained and tolerant cells, respectively. Subsequently, the cells were left for 5 days in RPMI medium with 10% human pooled serum and BrdU. Upon completion of this period, the amount of BrdU (as a parameter of endoreplication) was quantified by a colorimetric assay, and raw OD data were recorded. *N* = 6, Wilcoxon; ^*^p < 0.05; ^***^p < 0.001. **(B,C)** Human monocytes were incubated for 24 h in RPMI, β-glucan or LPS (the former being the control, the latter two the trained and tolerant cells, respectively). Afterwards, the cells were left for 4 days and then RNA sequencing was performed on these cells without further treatment. Data are expressed as reads per kilobase per million reads (RPKM). *N* = 3, Wilcoxon; ^*^p < 0.05; ^**^p < 0.01.

Additionally, extensive incorporation of BrdU was observed in trained human monocytes that were in contact with β-glucan for 24 h (Figure 2A). Contrarily, incubation with LPS, which induces immune tolerance [the opposite of TI 31], led to an incorporation of BrdU similar to that found in control cells. As demonstrated in mice, checkpoint kinase 1 (*CHEK1*) has an important upstream role during endoreplication (32). Hence, the expression of this kinase was evaluated in order to find out whether it was also produced during β-glucan-induced TI in human monocytes. Compared to control monocytes, β-glucan-treated cells displayed a 1.5-fold upregulation of *CHEK1*, while LPS-treated cells exhibited a 1.5-fold downregulation of its expression (Figure 2B).

Since *HNT* is a key factor in the endoreplication in *Drosophila* and in the mosquito TI, we asked whether the ortholog in mammals *RREB1* was also participating in TI in monocytes. We observed 1.5-fold upregulation of this gene in monocytes treated with β-glucan, whereas LSP did not modify the expression of this gene (Figure 2C).

### Immune gene amplification in the LSB-AA695BB mosquito cell line and human monocytes

**Figure 3:**
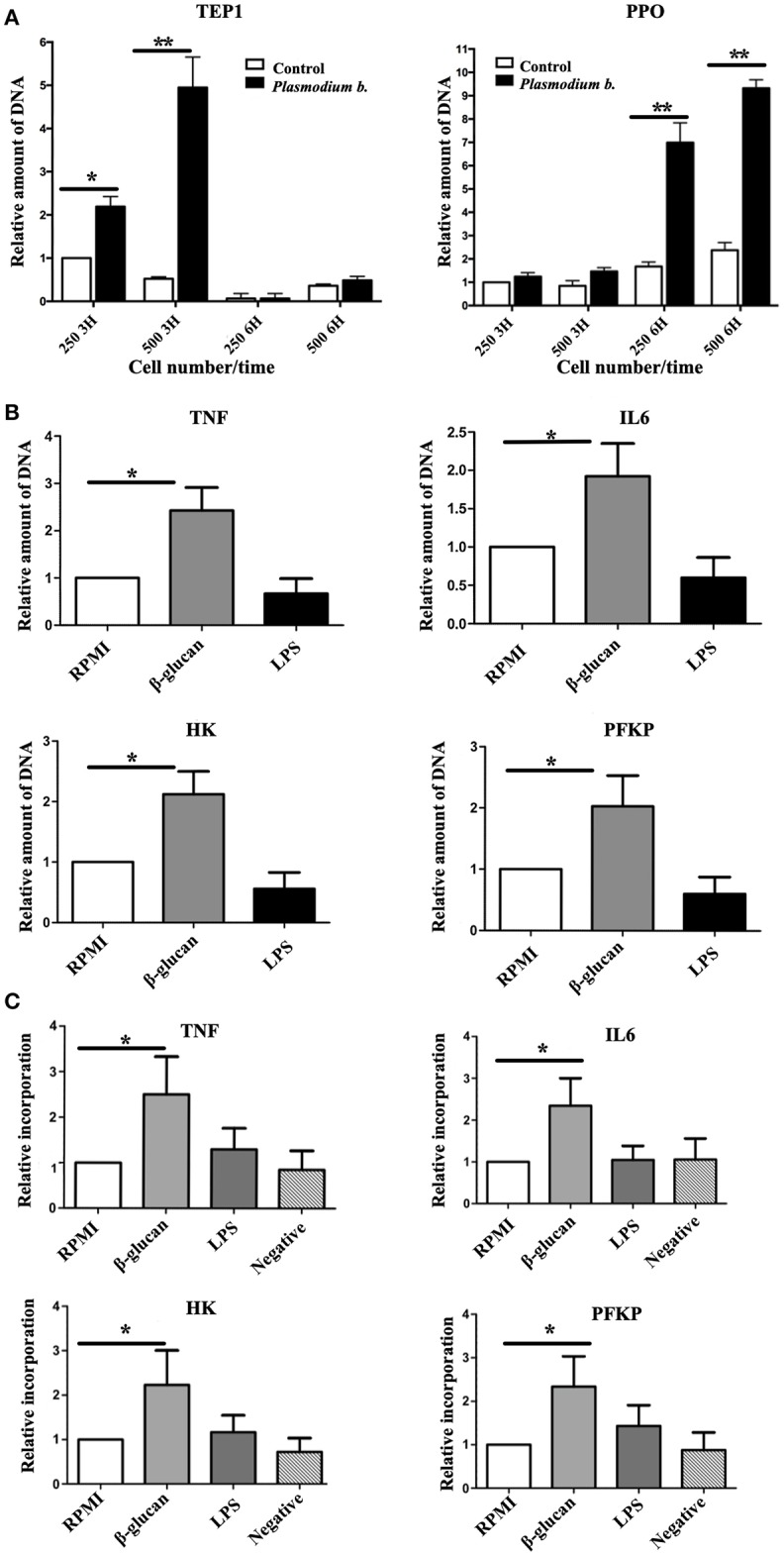
**(A)** The number of genomic copies of *TEP1* and *PPO1* following the exposure of 250,000 and 500,000 cells to *Plasmodium berghei* during 3 or 6 h. **(B)** Human monocytes were incubated for 24 h in RPMI, β-glucan or LPS (the former being the control, the latter two the trained and tolerant cells, respectively). Subsequently, the cells were left for 5 days in RPMI medium with 10% human pooled serum, and then harvested. The DNA was isolated and qPCR was run with primers for the promoter regions of *TNFA, IL6, HK*, and *PFKP*. Expression in the RPMI control group was set at 1. Relative amount of DNA of the trained (β-glucan)this and immunotolerant (LPS) groups was determined. *N* = 6, Wilcoxon; ^*^p < 0.05; ^**^p < 0.01. The data represent three and six different experiments. **(C)** Human monocytes were incubated for 24 h in RPMI, β-glucan or LPS (the former being the control, the latter two the trained and tolerant cells, respectively). Afterwards, the cells were left for 5 days in RPMI medium with 10% human pooled serum and BrdU, and then harvested and sonicated. DNA was incubated overnight with an anti BrdU antibody and beads. The next day unbound DNA (thus not containing BrdU) was washed away and qPCR was run with primers for the promoter regions of *TNA, IL6, HK*, and *PFKP*. The expression in the RPMI control group was set at 1. Relative expression in the trained (β-glucan) and immunotolerant (LPS) groups was determined. A negative control group was β-glucan-trained but not incubated with BrdU. *N* = 6, Wilcoxon; ^*^p < 0.05.

One characteristic of endoreplication is the increase in the number of copies of relevant genes. To determine whether there is an amplification in the immune response genes in TI, the LSB-AA695BB mosquito cell line was trained with Zymosan or the *Plasmodium* parasite extract. Two genes relevant to mosquito immunity constituted the main focus of the analysis: thioester binding protein (*TEP1*) and prophenoloxidase (*PPO1*). The expression of *CTL4, CTL6*, and *DNMT2* was also evaluated, but no differences existed between challenged and control cells (Supplementary Figure 1). However, the challenged group contained an elevated number of copies of *TEP1* and *PPO1*. While the former encodes for a C3-like complement protein that binds to malaria parasites in *An. gambiae* mosquitoes, the protein of latter gene melanizes parasites, and concomitantly produces reactive oxygen species (33). After exposure to *Plasmodium*, the greatest number of copies for *TEP*1 was observed at 3 h and for *PPO1* at 6 h (6 and 9 copies, respectively) (Figure 3A).

To determine whether endoreplication takes place in genes essential for TI in humans (34, 35), the number of copies of these genes was assessed 5 days after inducing TI or tolerance. A clear 2- to 3-fold increase in the expression of such genes was found in β-glucan-treated cells (Figure 3B). To further explore the endoreplication of these genes, cells were incubated with BrdU for 5 days, following 24 h of contact with RPMI (control), β-glucan (TI) or LPS (tolerance). The BrdU-incorporated DNA was isolated on day 6 and the relative amount (by setting the control group at 1) of promoter sequences of *TNFA, IL6, HK*, and *PFKP* was determined (Figure 3C). Compared to the control, there was clearly a greater BrdU incorporation at TI-linked promoter sites in the β-glucan-treated cells, indicating endoreplication.

### DNA synthesis is essential for the expression of TI markers in the LSB-AA695BB mosquito cell line and in human monocytes

For both the mosquito cell line and human monocytes, the possible participation of DNA synthesis in the establishment of TI was evaluated by cisplatin treatment. This compound is a potent antitumor agent that acts via cross-linking to DNA to form intra- and inter-strand adducts, thereby suppressing DNA synthesis (36). Cisplatin has been used successfully in insects to block DNA synthesis during the process of midgut repair in *Bombyx mori* (37).

**Figure 4:**
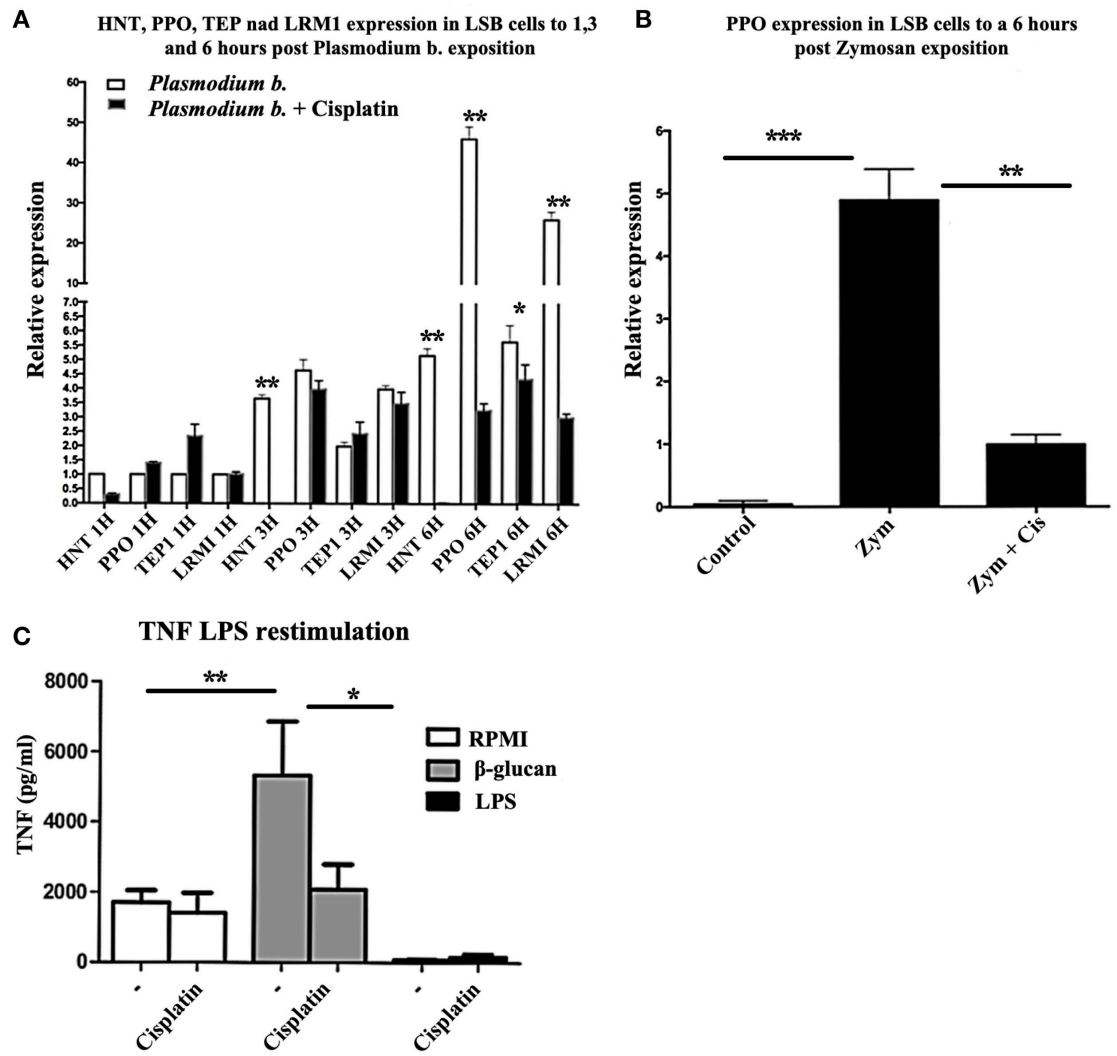
**(A)** Relative expression levels (CT) of *HNT, PPO1, TEP1*, and *LRIM1* in LSB cells at 1, 3, and 6 h of exposure to *Plasmodium berghei* or *Plasmodium berghei* + cisplatin. *N* = 3, Wilcoxon; ^*^p < 0.05; ^**^p < 0.01. The data are based on three experiments. **(B)** LSB cells were treated for 6 h with Zymosan alone or Zymosan + cisplatin and the relative expression of *PPO* was determined. *N* = 3, Wilcoxon; ^**^p < 0.01; ^***^p < 0.001. Values are the mean of three experiments. **(C)** Human monocytes were incubated for 24 h in RPMI, β-glucan or LPS (the former being the control, the latter two the trained and tolerant cells, respectively). Afterwards, the cells were left for 5 days in RPMI medium with 10% human pooled serum with or without cisplatin. Then cells were re-stimulated for 24 h with 10 ng/ml LPS. The production of TNFα in the supernatant was quantified by ELISA. *N* = 6, Wilcoxon; ^*^p < 0.05; ^**^p < 0.01.

In the mosquito cell line incubated with cisplatin, the expression of *HNT, PPO1, TEP1*, and *LRIMI* genes was reduced (Figures 4A,B) after 6 h of treatment with Zymosan or *Plasmodium* extract. The relative expression of *HNT* was eliminated and *PPO1* declined from 50 to 3-fold. When human monocytes were trained with β-glucan and a week later exposed to LPS, *TNFA* expression was induced, as expected. However, cisplatin treatment was also able to curb the expression of this gene, showing a clear inhibition of TI (Figure 4C).

## Discussion

The present results demonstrate that DNA synthesis is key during the process of stimulating TI in human monocytes and in the *Anopheles albimanus* mosquito cell line. The onset of TI brought an increase in DNA synthesis and a greater number of copies for genes related to immune markers. The blocking of DNA synthesis, on the other hand, prevented the establishment of TI.

It is likely that endoreplication is part of the mechanism of TI in human myeloid cells, as well as in mosquitoes and mosquito cell lines. For example, triggering the immune response in *An. albimanus* is accompanied by an intense DNA synthesis and formation of polytene chromosomes (22). The importance of endoreplication is also supported by the amplification of the purine synthesis pathway (a crucial step for DNA synthesis) during the induction of TI in human monocytes (34, 38). Inhibition of purine synthesis inhibited the induction of trained immunity (34, 39). Cisplatin treatment herein proved to have the same effect.

In the same way, during the induction of immune memory in *An. albimanus*, we have observed intensive DNA synthesis in the midgut and other tissues after priming with *P. berghei*. DNA synthesis is enhanced after a challenge with a large quantity of parasites (23). It is likely that cells of different tissues from entering endoreplication in which multiple copies of the genome or amplicons can be made unless the cell enters mitosis or proliferation (40).

In the priming of *An. albimanus* we observed the overexpression of the hindsight gene (*HNT)*, which is involved in the change of the cell cycle to endoreplication in *Drosophila* (26). Interestingly, *HNT* is overexpressed in response to infection with the malaria parasite *P. berghei* (23). The same occurs in the case of *Aedes aegypti* infected with dengue virus. When DNA synthesis is blocked by cisplatin, dengue virus is able to replicate in the mosquito midgut (24, 25). Hence, midgut cells apparently require the amplification of certain genes, such as *TEP1* or *PPO1* (but not *CTL*s), for a fast and effective immune response. These genes, in turn, likely participate in the rapid production of effector transcripts and proteins (27). The genetic information for such production may be stored in the copies of key genes responsible for a rapid response to a second insult. DNA synthesis and the formation of polytene chromosomes could be mechanisms for increasing the activity of genes that synthesize large amounts of immune defense proteins (23).

We have observed similar results with *Aedes aegypti* and the dengue virus, including intensive DNA synthesis, activation of the Notch pathway, and overexpression of Delta and Notch (the ligand and receptor of the Notch pathway), and *HNT* (25). Additionally, by blocking DNA synthesis with cisplatin, which diminishes the overall effects of the transcriptional machinery through DNA abduct formation, the memory effect is eliminated in both *An. albimanus* and *Aedes aegypti* mosquitoes. This indicates that DNA synthesis and endoreplication are part of the mechanisms of memory (24, 25). Cisplatin treatment causes the same outcome in monocytes and mosquito cell lines, reducing the expression of the immune response genes related to innate immune memory.

Cisplatin is a known anti-cancer agent, preferentially binding to the guanine base. It interferes with DNA replication by forming cross-linked DNA adducts, thereby suppressing DNA synthesis. At high doses of cisplatin (100 mM), inhibition of DNA replication leads to apoptosis in a human cancerous cell line (41). At low doses (100 μM), it inhibits DNA duplication in *Bombyx mori* (37). Since low doses were used in the current study, the effect was probably on DNA duplication. However, it is also possible that apoptosis was activated. No apoptotic cells were detected herein, but further experiments are required to confirm the present findings. Nevertheless, to support the endoreplication specificity in TI we need abolishing genes involved in this process such as CDK. We are currently silencing genes involved in the Notch pathway (24), the cell cycle and endoreplication to better understand the molecular mechanisms.

The generation of essential immune molecules may be fostered by the amplification of genes, which leads to an increase in the number of templates available for transcription. By amplifying the number of copies of genes, the mosquito epithelial cells, and macrophages can store the information necessary for a rapid and efficient production of the RNA and proteins required to respond to a second challenge with the same or another pathogen. This mechanism avoids the cost of cell proliferation represented by vertebrate immune memory.

The amplification of *HNT* and *RBB1* requires more in-depth study to determine the different steps that occur during the activation of the Notch pathway in monocytes and mosquito cells. Moreover, the relevance of endoreplication in the establishment of innate immune memory needs to be further addressed *in vivo* in vertebrates.

In *Arabidopsis*, overexpression was reported for the *OSD1, UV14*, and *CPR5* genes involved in cell cycle regulation and immunity in plants (42, 43). *OSD1* and *UV14* are negative regulators of APC/C, which is responsible for degrading cell cycle proteins. The function of *OSD1* and *UV14* brings about various defects in cell cycle progression, including the omission of cell division in meiosis and greater endoreplication. The overexpression of these genes enhances resistance against virulent bacterial pathogens via upregulation of disease resistance (R) genes, which encode plant immune receptors that recognize effector proteins secreted from pathogens, activate R proteins that generate transcriptional reprogramming, and often program cell death to inhibit the spreading of pathogens in plants. It is not clear whether this mechanism was operating in the present study. Therefore, we are currently looking for CDK-cyclin complexes and homolog genes involved in endoreplication and the overexpression of innate immune molecules.

In conclusion, evidence is herein provided of endoreplication as a possible factor in the establishment of TI in an *Anopheles* cell line and *in vitro* in human monocytes. Endoreplication appears to be a well-conserved mechanism throughout evolution in innate immune memory.

## Author contributions

JC-C, RA, MN, and HL-M conceived and designed the experiments. JC-C, RA, and VV-P performed the experiments. JC-C, RA, RM-T, and SH-M analyzed the data. BR-T and FC-P contributed reagents and material. JC-C, RA, MN, and HL-M wrote the paper. All authors read and approved the final version of the manuscript.

### Conflict of interest statement

The authors declare that the research was conducted in the absence of any commercial or financial relationships that could be construed as a potential conflict of interest.
