# Supplementary figures and images for: DNA Synthesis Is Activated in Mosquitoes and Human Monocytes During the Induction of Innate Immune Memory
Source: Front Immunol. 2018 Nov 30;9:2834. doi: 10.3389/fimmu.2018.02834 (PMC6284063; doi:10.3389/fimmu.2018.02834)

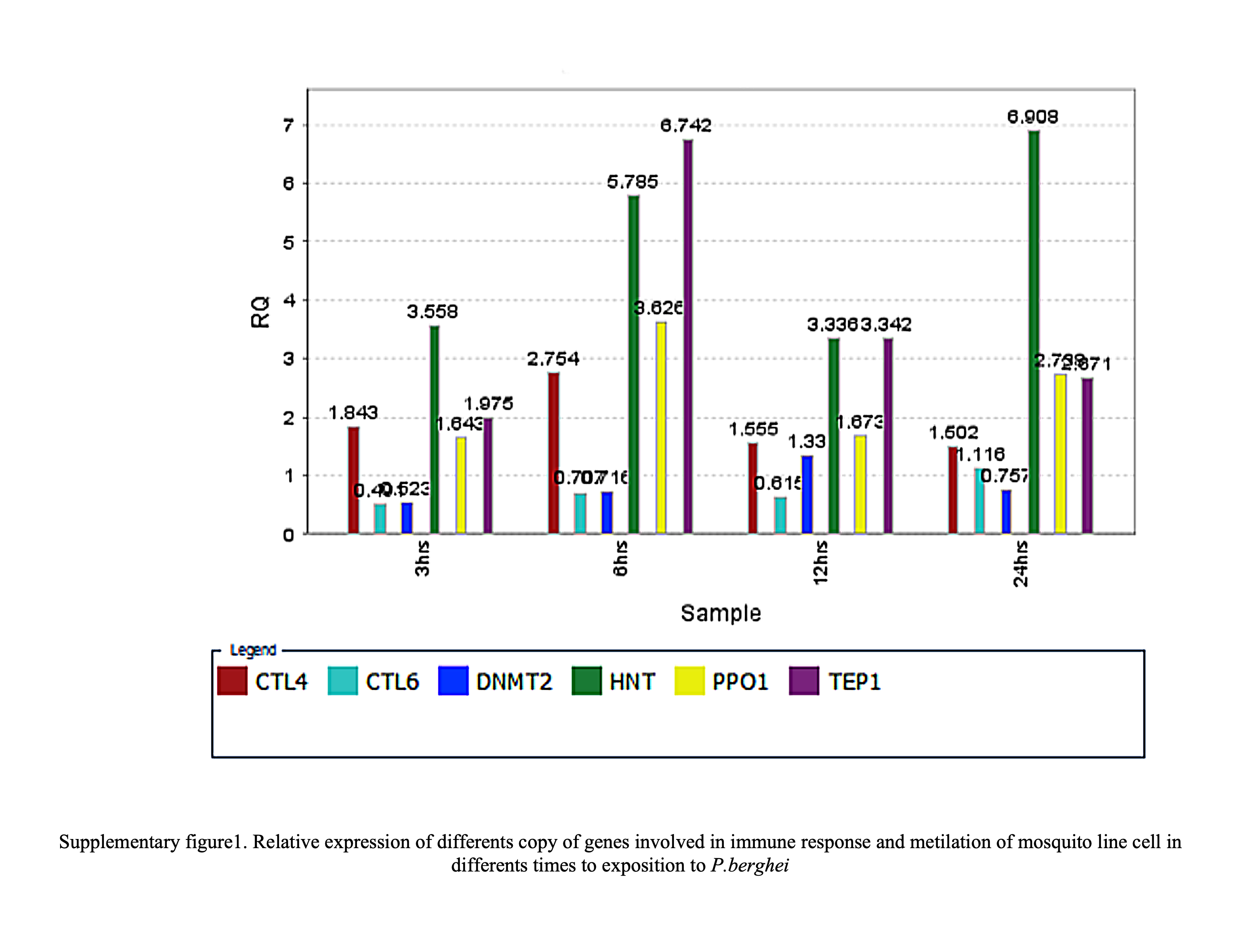

Supplement: Supplementary file 1 [file Image_1.TIFF]
